# Borrelia burgdorferi: A Rare Cause of Stroke

**DOI:** 10.7759/cureus.77061

**Published:** 2025-01-07

**Authors:** João Filipe Félix Vieira Afonso, Mafalda Maria Santos, Joana Vieira, Rafael Oliveira, Ana Filipa Rodrigues

**Affiliations:** 1 Internal Medicine Department, Unidade Local de Saúde do Oeste, Caldas da Rainha, PRT

**Keywords:** borrelia burgdorferi infection, cerebral vasculitis, internal medicine in rural areas, ischemic stroke, lyme neuroborreliosis

## Abstract

Lyme disease is the most common tick-borne zoonosis in Europe. It is a multisystemic infectious disease that can produce cerebrovascular events on rare occasions. Here, the authors describe a case of a 58-year-old female patient living in a rural area who presented to the hospital with dysarthria, right central facial paralysis, right hypoesthesia, and severe right hemiparesis. Imaging revealed an infarct in the left corona radiata, posterior limb of the internal capsule, posterior parietal region, and multiple microhemorrhages scattered throughout the cerebral hemispheres. She was hospitalized with the diagnosis of ischemic stroke. Given the absence of known risk factors, the diagnostic workup included a lumbar punction, which showed cerebrospinal fluid serology positive for immunoglobulin M against *Borrelia burgdorferi*. The patient was treated with doxycycline for 21 days, leading to clinical improvement.

## Introduction

Stroke is a serious neurological condition caused by disruption in cerebral blood flow, either due to ischemia or hemorrhage. Common ischemic causes include thrombosis, embolism, systemic hypoperfusion, and blood disorders. Nevertheless, in patients without risk factors, rarer causes, such as infectious diseases, should be considered. Lyme neuroborreliosis, caused by *Borrelia burgdorferi*, can lead to cerebral vasculitis and stroke, particularly in endemic areas and in patients without traditional cardiovascular risk factors.

Lyme disease is the most common tick-borne zoonosis in Europe and the United States of America [1]. It is a multisystem infectious disease that progresses through three phases: early localized phase (from the first days up to a month after the tick bite, and it is typically characterized by erythema migrans), early disseminated phase (weeks to months after the bite, and some of the possible manifestations include cardiac, neurological, hepatic, renal, ocular, cutaneous, musculoskeletal, and lymphadenopathy), and late phase (months to years after the bite, in which musculoskeletal, neurological, and cutaneous manifestations can appear) [1]. Systemic symptoms such as fatigue, fever, anorexia, and headache may persist throughout the disease. Central nervous system involvement occurs in 10%-15% of cases [1], with an incidence of 2.6 cases per 100,000 inhabitants [2]. Symptoms typically appear 1-12 weeks after the tick bite [3].

Early Lyme neuroborreliosis (symptom duration less than six months) accounts for over 95% of cases [3]. In Europe, the most common manifestation is meningoradiculitis [3]. Rarely, Lyme neuroborreliosis may involve vasculitic processes, potentially causing cerebrovascular events such as transient ischemic attack and ischemic or hemorrhagic stroke [4]. Cerebrospinal fluid (CSF) analysis, showing pleocytosis and elevated protein levels, is essential for diagnosis. The gold standard for diagnosis is the intrathecal detection of antibodies against *B. burgdorferi* [3]. Late Lyme neuroborreliosis may present with chronic encephalomyelitis, radiculoneuritis, meningitis, peripheral neuropathy, and stroke-like symptoms due to vasculitis and cerebral infarction [3,5-8]. Its diagnosis requires serology testing in both blood and CSF, as they are usually positive [3,5,8,9]. Lymphocytic pleocytosis with moderate protein elevation and normal glucose levels is frequently observed in CSF [3,5,8]. Imaging findings may show inflammatory areas with increased T2 and fluid-attenuated inversion recovery signal [7].

According to the European Federation of Neurological Societies (EFNS) guidelines, a definitive diagnosis of Lyme neuroborreliosis requires the presence of three criteria: neurological symptoms suggestive of Lyme neuroborreliosis not caused by other obvious reasons, CSF pleocytosis, and the presence of antibodies in CSF against *B. burgdorferi* (with intrathecal production) [3]. The diagnosis is considered possible if only two of the mentioned criteria are present. Recommended treatment includes oral doxycycline or intravenous ceftriaxone for 14 days in early disseminated forms with neurological involvement and intravenous ceftriaxone for 21 days in late-phase cases [3]. Serologies are not used to assess treatment response, as they may remain positive for months [3,5,7,9].

## Case presentation

A 58-year-old woman living in a rural area presented to the emergency department with right-sided paresthesia and decreased muscle strength for more than six hours. She had no significant medical history and no known risk factors.

**Figure 1:**
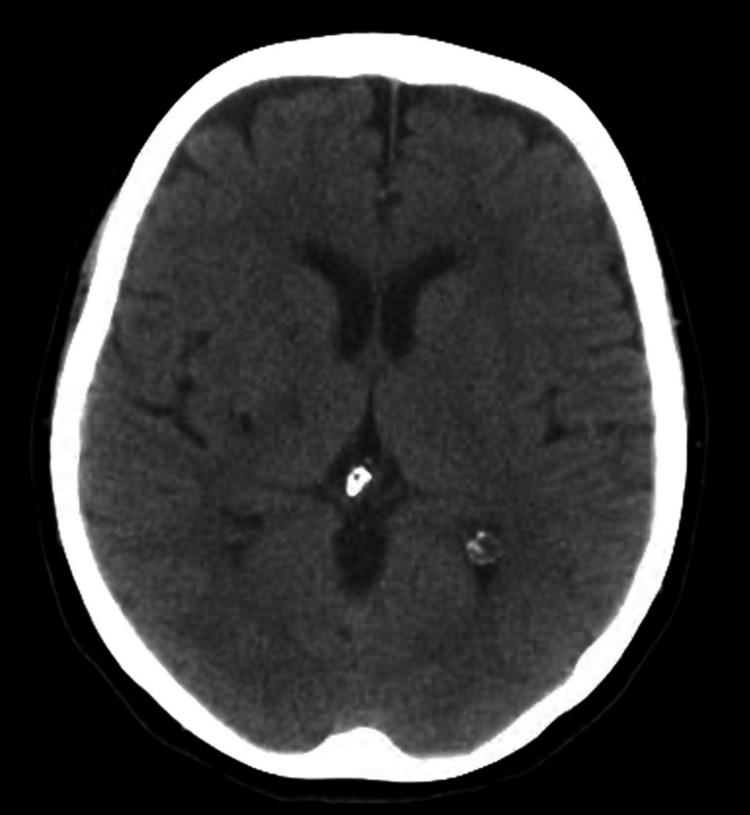
Axial view of the cerebral CT scan (noncontrast), showing no ischemic lesions CT: computed tomography

On admission, she had a Glasgow Coma Scale of 15 (eye response 4 points, motor response 6 points, and verbal response 5 points). Her vital signs included blood pressure 134/75 mmHg, heart rate 96 beats per minute, SpO_2_ 98% (room air), and temperature 36.6°. Neurological examination revealed mild dysarthria without aphasia, right central facial paralysis, right hypoesthesia, and severe right hemiparesis, with a score of 9 on the National Institutes of Health Stroke Scale. No meningeal signs were observed with Brudzinski and Kernig tests. Brain and supra-aortic computed tomography (CT), with contrast, revealed no ischemic areas or intra-arterial thrombosis/stenosis (Figure 1). After consultation with the neurology service, stroke pathway activation was not warranted.

She was admitted for further study and started on antiplatelet therapy (aspirin 100 mg) and high-potency statin (atorvastatin 80 mg). During hospitalization, the history of the current illness became of utmost importance: the patient reported fatigue, migratory polyarthralgia, morning stiffness for more than 30 minutes, and headaches for several weeks. She mentioned having dogs and living in the countryside but could not recall any tick bites.

**Figure 2:**
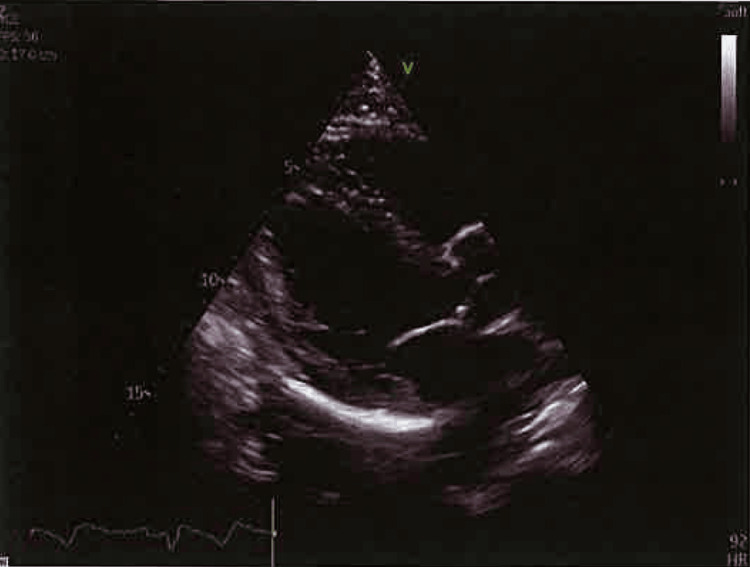
Echocardiogram, showing no signs of endocarditis

Diagnostic studies included sinus rhythm on electrocardiogram and a normal echocardiogram without any images suggestive of endocarditis (Figure 2). Laboratory tests (Table 1) showed normal values for glycated hemoglobin, total cholesterol, and low-density lipoproteins. The only notable change was an elevated erythrocyte sedimentation rate. Extended workup was pursued due to the absence of cardiovascular risk factors. Autoimmune (antinuclear antibodies, anti-Sjögren's-syndrome-related antigen A/Sjögren's-syndrome-related antigen B, rheumatoid factor, anticitrullinated protein, antidouble-stranded DNA, antiphospholipid antibodies, and antineutrophil cytoplasmic antibodies), thrombophilia panels (functional levels of protein C and S, von Willebrand factor, prothrombin antibody, and homocysteine), HIV, syphilis, hepatitis C, and interferon-gamma release assay test were negative.

**Table 1:** Laboratory and lumbar punction results CRP: C-reactive protein; LDL: low-density lipoprotein; HDL: high-density lipoprotein; ESR: erythrocyte sedimentation rate

Parameter	Patient value	Normal range
White blood count	7.9 × 10^3^ u/L	4.0-10.0 × 10^3^ u/L
Hemoglobin	14.2 g/L	13.6-18.0 g/L
CRP	0.3 mg/dL	<0.5 mg/dL
Total cholesterol	180 mg/dL	<200 mg/dL
LDL	100 mg/dL	<130 mg/dL
HDL	44 mg/dL	>60 mg/dL (negative risk for cardiac disease)
Triglycerides	180 mg/dL	<150 mg/dL
ESR	43 mm/hour	12-20 mm/hour
Creatinine	0.71 mg/dL	0.6-1.1 mg/dL
Urea	33 mg/dL	21-43 mg/dL
Glycated hemoglobin	5.4%	4%-6%
Uric acid	4.5 mg/dL	2.6-6 mg/dL

**Figure 3:**
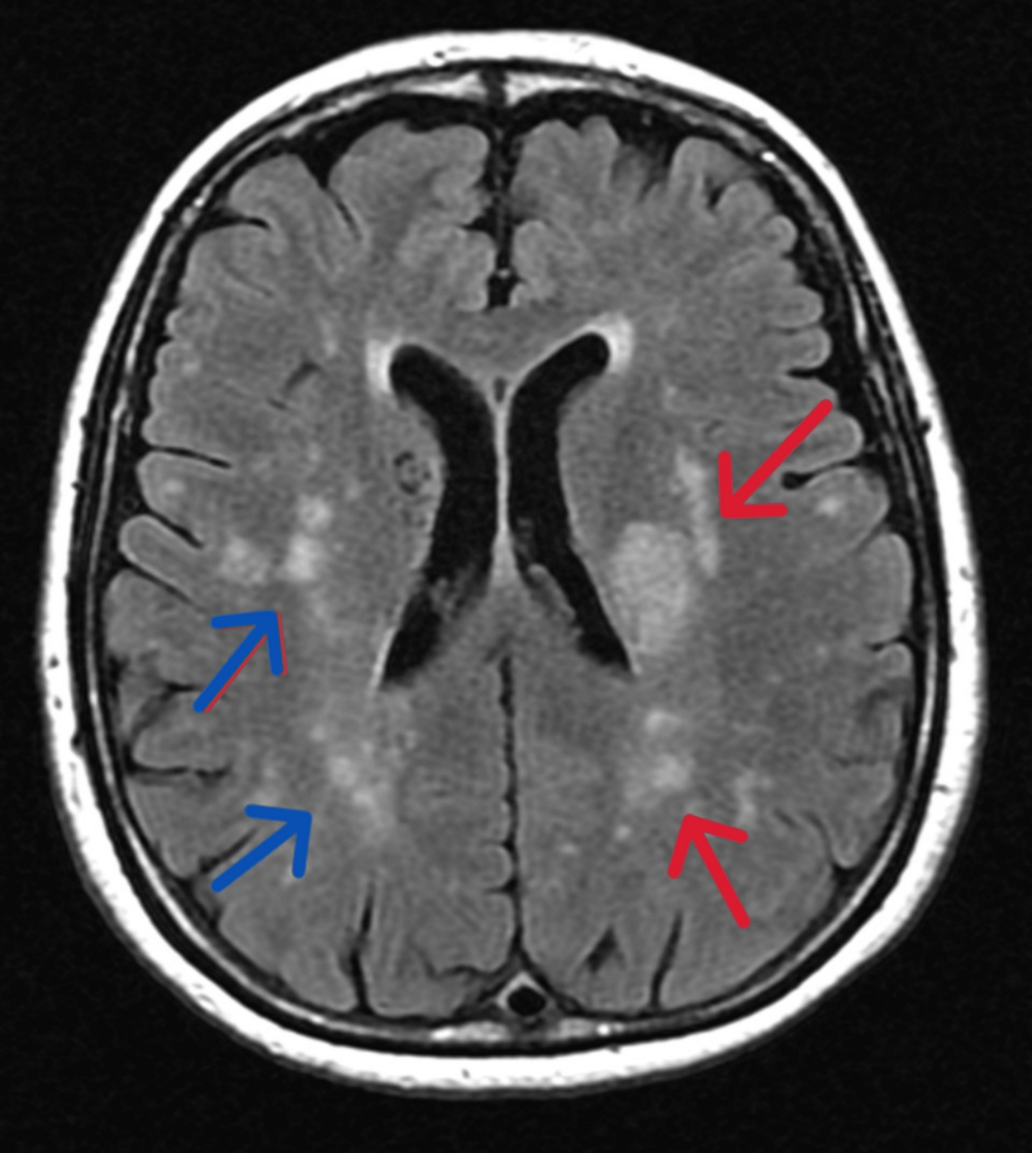
Axial view of cerebral MRI (T2 FLAIR). The red arrows (left hemisphere) show an area of restricted diffusion in the left corona radiata, posterior limb of the internal capsule, and small acute parietal lesion. The blue arrows (right hemisphere) reveal microvascular ischemic lesions MRI: magnetic resonance imaging; FLAIR: fluid-attenuated inversion recovery

**Figure 4:**
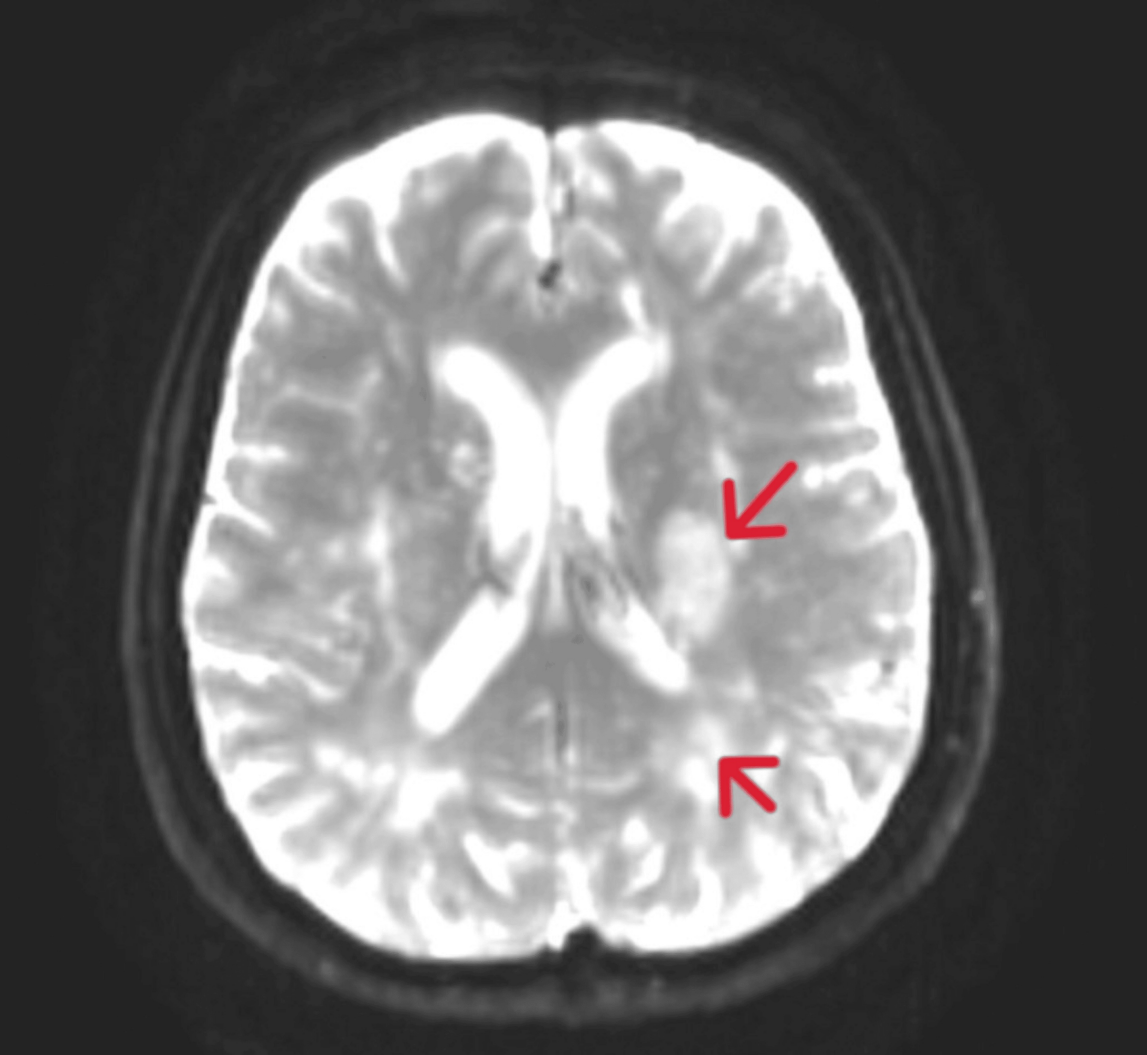
Axial view of cerebral MRI (DWI). The red arrows show the acute ischemic lesion MRI: magnetic resonance imaging; DWI: diffusion-weighted imaging

**Figure 5:**
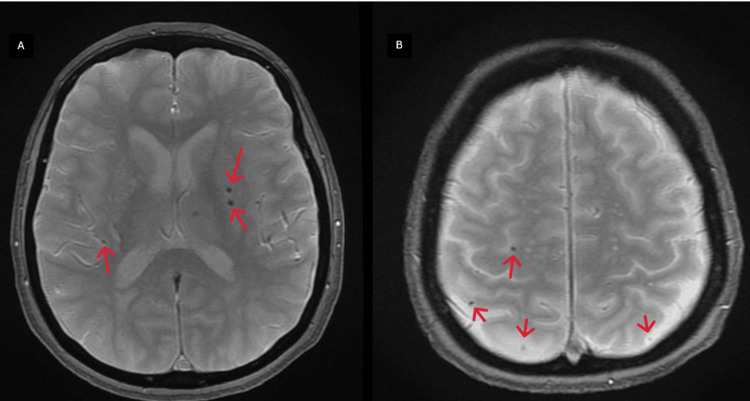
Axial view of cerebral MRI (T2*). (A,B) The bilateral microhemorrhages at different levels (red arrows) MRI: magnetic resonance imaging

Brain magnetic resonance imaging (MRI) showed an acute area of restricted diffusion in the left corona radiata, the posterior limb of the internal capsule, a subcortical lesion in the posterior parietal region, microvascular ischemic lesions, and multiple microhemorrhages scattered throughout the cerebral hemispheres (Figures 3-5).

She was discharged after seven days pending results for infectious causes of cerebral vasculitis, maintaining mild dysarthria, right central facial paralysis, right hypoesthesia, and right hemiparesis grade 3.

At follow-up, her deficits improved, but headaches and paresthesia persisted. Tests for herpes virus, varicella-zoster virus, Epstein-Barr virus, and cytomegalovirus were negative, but immunoglobulin M (IgM) was positive for *B. burgdorferi*. After those results, a lumbar puncture (Table 2) was performed, revealing 20 cells, predominantly lymphocytes (95%), 70 mg/dL of proteins, and IgM positive for *B. burgdorferi*. She was treated with doxycycline (100 mg twice daily) for 21 days, leading to negative IgM and a positive immunoglobulin G on revaluation. Six months after treatment, the patient had clinical improvement, being capable of walking and talking without any major restrictions.

**Table 2:** Lumbar punction results

Lumbar punction	Patient value	Normal range
Cells	20/uL	-
Proteins	70 mg/dL	15-45 mg/dL
Glucose	63 mg/dL	40-70 mg/dL

## Discussion

Lyme neuroborreliosis is a rare cause of stroke (<1%) [6]. It has been especially described in European case reports [10,11]. The proposed mechanism of ischemic stroke due to Lyme neuroborreliosis is secondary to localized inflammatory vasculitis.

There is no evidence for testing Lyme disease in every patient with an ischemic stroke and known cardiovascular risk factors. However, this should be considered when a patient comes from an endemic area when no other evident cause of stroke is found and in the case of multiterritorial strokes or radiological signs of vasculitis [4,6]. It can present as hemorrhagic or ischemic strokes. In parenchymal brain imaging, multiple territory strokes can be observed. However, negative radiological imaging studies do not exclude the diagnosis, as it involves small vessels. Our patient presented a main ischemic lesion on the left hemisphere accompanied by microvascular ischemic lesions and microhemorrhages bilaterally, compatible with vasculitis, and these were only observed in MRI. The patient did not have any cardiovascular risk factors, so an extended study was performed. CSF analysis with the presence of *B. burgdorferi *antibodies is the “gold standard” for the diagnosis. A thorough history of tick exposure or systemic symptoms in the previous weeks to months must be obtained in suspicious cases [3]. The patient's CSF revealed a lymphocytic pleocytosis with the presence of *B. burgdorferi* IgM.

Our case of Lyme neuroborreliosis presenting as stroke illustrates the importance of considering rare etiologies in younger patients without any significant cerebrovascular risk factors, especially in endemic areas such as ours. According to the EFNS diagnostic criteria, our patient had a definite Lyme neuroborreliosis [8]. About 88% of the patients show clinical improvement in neurological symptoms with treatment [11]. She underwent treatment with doxycycline for 21 days, showing important residual symptoms with functional limitation (dysarthria and hemiparesis), which improved mainly thanks to physiotherapy, showing that Lyme neuroborreliosis can lead to long-term sequels, such as sensory disorders and extremity paresis [3,4,11].

## Conclusions

In high-prevalence areas and patients without major risk factors, other causes of stroke, such as Lyme neuroborreliosis, should be considered. The clinical history, including epidemiology and prodromal symptoms, plays a crucial role in directing the diagnosis. Imaging studies, such as CT scans or MRIs, and antibody detection in CSF, play an important role in the diagnosis of this disease. This case underlines the importance of a detailed medical history in order to think in the rarest causes of stroke.
